# Comparison of fine needle aspiration of thyroid nodules and lymph nodes with 22- and 25-gauge needles: a retrospective study

**DOI:** 10.1097/MS9.0000000000003664

**Published:** 2025-07-30

**Authors:** Mohammad Yahia Alzahrani, Mohammad Sayed Abdelghaffar, Tariq Hashim Adlan

**Affiliations:** Department of Interventional Radiology, King Fahad Specialist Hospital, Dammam, Saudi Arabia

**Keywords:** 22-gauge needle, 25-gauge needle, cytology, lymph nodes, thyroid nodules, ultrasound-guided fine-needle aspiration biopsy

## Abstract

**Background:**

Approximately 15% of fine-needle aspiration biopsy (FNAB) procedures are considered inconclusive, caused by various factors such as nodule characteristics, level of expertise, and needle size. Since needle size may play a crucial role in both adequacy of FNAB samples and reduction of complications, our objective was to compare cytology specimen adequacy, blood contamination, and findings in thyroid nodules and lymph nodes using 22- and 25-gauge needles in fine-needle aspiration (FNA).

**Materials and methods:**

A retrospective comparative cohort study was conducted at King Fahad Specialist Hospital, Saudi Arabia. Data were collected from electronic medical records between January and December 2021. An ultrasound-guided FNA procedure was performed. Each nodule was sampled in three passes using a 25-gauge and a 22-gauge fine needle. A total of 153 (115 with thyroid nodules; 57 with 22-gauge and 58 with 25-gauge and 38 with lymph nodes; 21 with 22-gauge and 17 with 25-gauge) cases were included. Data analysis was done using SPSS version 26. All cases were divided into two groups: 22-gauge or 25-gauge needle group. Chi-square test and binary logistic regression were used for comparison.

**Results:**

No significant differences were found. Adequacy rates were 93.9% for thyroid and 97.4% for lymph node samples (*P* = 0.68). Conclusive diagnoses were 97.4% for thyroid and 94.7% for lymph nodes (*P* = 0.137). Bloody samples were more frequent in lymph nodes (23.7%) than thyroid (11.3%) (*P* = 0.111). Needle size did not affect adequacy, but the number of passes was a significant predictor (odds ratio: 0.068, 95% confidence interval: 0.008–0.570, *P* = 0.013).

**Conclusion:**

The study highlights the importance of the number of passes during the procedure, with a higher number of passes associated with decreased odds of obtaining an adequate sample. The needle size does not significantly impact the various variables assessed.

## Introduction

Thyroid nodules are defined as localized enlargements of the thyroid gland. They are quite common, with a high clinical prevalence estimated at 3–7% in the general population, and they tend to occur more frequently in females^[1,2]^. Nowadays, nearly half of all nodules are detected through imaging techniques. Ultrasound has proven to be particularly effective in identifying thyroid nodules, with a prevalence about 10 times higher than that determined through clinical examination^[3]^.

The utilization of ultrasound guidance in fine-needle aspiration biopsy (FNAB) provides excellent sensitivity and specificity when detecting malignant thyroid nodules prior to surgical removal^[4]^. This technique has been employed since the 1930s, when the first report on FNAB’s application to thyroid nodules was published^[5]^. Since then, medical professionals have endeavored to devise approaches for obtaining larger amounts of aspirated material. The incorporation of US guidance in FNAB has significantly decreased the occurrence of inconclusive aspirations, making it the preferred method for evaluating thyroid nodules^[6]^. The sensitivity of ultrasonography in assessing metastatic lymph nodes in the neck is low. Ultrasound-guided fine needle aspiration is an effective method for preoperative diagnosis of suspected lymphadenopathy. The dimensions of the lymph node significantly affect FNAB sampling. It was proposed that US-FNAB is suitable for lymph nodes measuring ≥10 mm^[7]^, but several researchers advocated for US-FNAB in patients with lymph nodes ≥5 mm^[8]^.

**HIGHLIGHTS**

- This is a retrospective study conducted to compare the adequacy rate of cytology specimens of thyroid nodules and lymph nodes obtained by 22- and 25-gauge needles.
- There was no significant difference in terms of adequacy, bloody samples, conclusiveness, or the number of passes made during the FNA procedure between the two needle sizes (22 and 25).
- A higher percentage of lymph node samples (78.9%) required more than three passes compared to thyroid samples (60%) with a statistical significance of *P* = 0.05.
- Needle size 22 had higher incidence of bloody sample (*P* = 0.002).
- The number of passes was a significant predictor of adequacy, with an OR of 0.068 (95% CI: 0.008–0.570) and a *P* value of 0.013.

Ultrasound-guided FNAB is generally a safe procedure with minimal morbidity, although some patients may experience localized pain and hemorrhage^[9]^. These complications are directly associated with the size of the needle utilized.

Additionally, even when performed by experienced physicians, approximately 15% of FNAB procedures are considered inconclusive^[10]^. Various factors contribute to these unsuccessful procedures, such as nodule characteristics, the pathologist’s level of expertise, and the needle size used^[11]^. Presently, several needle diameters, spanning from 21 to 27 gauge, are employed in US-guided biopsies, utilizing varied techniques such as aspiration and capillary collection when appropriate^[12]^. Despite numerous advancements in cancer diagnosis, a standardized protocol for the size and type of needle for sample that facilitates optimal diagnosis while minimizing problems in the assessment of cervical lymph nodes remains absent. Given that needle size may play a crucial role in both the adequacy of FNAB samples for cytologic diagnosis of thyroid nodules and lymph nodes and the reduction of complications, our objective was to compare the adequacy rate of cytology specimens and to assess the blood contamination and conclusive or non-conclusive findings of cytology specimens of thyroid nodules and lymph nodes obtained by 22- and 25-gauge needles in fine-needle aspiration (FNA).

## Methodology

### Study design

A single-center retrospective comparative cohort study was conducted at to compare the adequacy rate of cytology specimens of thyroid nodules and lymph nodes obtained by 22- and 25-gauge needles in FNA. This study has been reported in line with the STROCSS criteria^[13]^.

### Study settings and patients

Patients who underwent FNA for thyroid nodules or lymph nodes using either 22-gauge or 25-gauge (22-gauge; 21GX1 1/2 RB GA and 25-gauge; 25GX11/2 RB GA, Nipro, Japan) were included. Patients who underwent FNA for reasons other than thyroid nodules or lymph nodes or patients who underwent FNA for a thyroid nodule or lymph nodes using a gauge other than 22 or 25-gauge were excluded from the study.

RaoSoft, an online sample size calculator, was used to calculate the sample size from an approximate population of 253 samples of FNA, according to a 5% margin of error, 80% power of the study, and 95% confidence level. The sample size was calculated to be 153 samples. Overall, 153 cases were included in the study.

### Data collection

Data on patients who underwent FNA of thyroid nodules or lymph nodes were retrospectively obtained from electronic medical records between January and December 2021. The extracted data included procedure details such as the needle gauge used (22-gauge or 25-gauge), number of passes performed (≤3, >3), and site of FNA (thyroid nodule or lymph node). Cytological findings included the adequacy of specimens (categorized as adequate if sufficient cells were present for diagnosis and inadequate if insufficient cells were obtained), presence of blood contamination (classified as bloody if visible blood affected the sample or non-bloody if the sample was clear), and diagnostic outcome (conclusive if a definitive benign or malignant diagnosis was made or non-conclusive if the sample did not allow for a definitive diagnosis). The extracted data from the electronic health records were stored in a pre-designed primary sheet.

### Procedures

Ultrasound-guided FNA procedure was performed by an experienced radiologist. Target nodules were selected according to the guidelines of the European Thyroid Association and the American Thyroid Association for the management of thyroid nodules and differentiated thyroid cancer. Each nodule was sampled in three passes using a 25-gauge fine needle with a length of 37 mm and a 22-gauge fine needle with a length of 31 mm. Punctuation procedures were performed with local anesthesia. The ultrasound probe position was adjusted so that the ultrasound beam was parallel to the fine needle to guide puncture and biopsy in real time. When the fine needle reached the target place of the nodule, the operator removed the stylus and moved the needle back and forth until the sample material was noticed at the hub of the needle.

Cytological analysis: extrusions of sample material obtained by FNA were placed on glass slides. Smears were fixed in alcohol and stained with hematoxylin and eosin staining. An experienced cytologist assessed each smear based on the scoring system. Specimen adequacy was determined according to the Bethesda System for Reporting Thyroid. Specimens with an abundance of colloid or a high number of inflammatory cells were considered adequate. On the contrary, specimens that only consisted of cystic contents were considered inadequate.

### Statistical analysis

The data were analyzed using the statistical software SPSS version 26. Categorical variables were presented as frequencies and percentages. All cases were divided into two groups based on the size of the needle used (22-gauge needle group versus 25-gauge needle group), and a comparison of both groups was conducted using a chi-square test. Additionally, binary logistic regression was used. A *P* value of <0.05 was considered statistically significant. The references were managed using EndNote 8 reference management software.

### Ethical statement

The confidentiality of the patients was maintained. Approval for this research was received from the Institutional Review Board of the hospital where the research was conducted. This study was also registered in the Research Registry with research registry UID.

## Results

Out of 115 thyroid cases, adequate results were obtained in 108 out of 115 patients (93.9%); bloody samples were observed in 13 patients (11.3%); non-bloody samples were found in 102 patients (88.7%); conclusive results were obtained in 112 patients (97.4%); and non-conclusive results were found in only 3 patients (2.6%). The number of passes during the FNA procedure was recorded and categorized into two groups: patients who received three or fewer passes (69 patients, 60%) and those who received more than three passes (46 patients, 40%) (Table 1).

**Table 1:** Frequency of all variables in patients with thyroid

Variables		Thyroid (n = 115)
		N (%)
Adequate		108 (93.9)
Inadequate		7 (6.1)
Bloody		13 (11.3)
Non-bloody		102 (88.7)
Conclusive		112 (97.4)
Non-conclusive		3 (2.6)
No. of passes	≤3	69 (60)
	>3	46 (40)

Out of 38 lymph node cases, adequate results were obtained in 37 out of 38 patients (97.4%), and inadequate results were obtained in only 1 patient (2.6%). Bloody samples were found in 9 patients (23.7%), while non-bloody samples were found in 29 patients (76.3%). Conclusive results were obtained in 36 patients (94.7%), while non-conclusive results were found in only 2 patients (5.3%). Thirty patients received three or fewer passes (78.9%), and eight patients received more than three passes (21.1%) (Table 2).

**Table 2:** Frequency of all variables in patients with lymph node

Variables		Lymph node (n = 38)
		N (%)
Adequate		37 (97.4)
Inadequate		1 (2.6)
Bloody		9 (23.7)
Non-bloody		29 (76.3)
Conclusive		36 (94.7)
Non-conclusive		2 (5.3)
No. of passes	≤3	30 (78.9)
	>3	8 (21.1)

In terms of sample adequacy, there was no significant difference between the two sample groups while 93.9% of thyroid samples and 97.4% of lymph node samples were considered adequate. Similarly, there was no significant difference between the two groups in terms of conclusiveness. 97.4% of thyroid samples and 94.7% of lymph node samples provided a conclusive diagnosis. However, there was a non-significant trend toward a higher incidence of bloody samples in the lymph node group (23.7%) compared to the thyroid group (11.3%). Additionally, there was a significant difference between the two groups in terms of the number of passes during the procedure. A higher percentage of lymph node samples (78.9%) required more than three passes compared to thyroid samples (60%) (Table 3).

**Table 3:** Comparison of thyroid vs lymph node group with respect to all variables

Variables		Thyroid	Lymph node	*P* value
		N (%)	N (%)	
Adequate		108 (93.9)	37 (97.4)	0.680
Inadequate		7 (6.1)	1 (2.6)	
Bloody		13 (11.3)	9 (23.7)	0.111
Non-bloody		102 (88.7)	29 (76.3)	
Conclusive		112 (97.4)	36 (94.7)	0.137
Non-conclusive		3 (2.6)	2 (5.3)	
No. of passes	≤3	69 (60)	30 (78.9)	**0.05**
	>3	46 (40)	8 (21.1)	

P value <0.05 is statistically significant.

Table 4 indicates that there was no significant difference in terms of adequacy, bloody samples, conclusiveness, or the number of passes made during the FNA procedure in thyroid samples between the two needle sizes (22 and 25) (*P* > 0.05). However, there was a slightly higher percentage of bloody samples and conclusive results in needle sizes 22 and 25, respectively, and a slightly higher percentage of samples requiring more than three passes with needle size 22. Nevertheless, these differences were not statistically significant (Table 4).

**Table 4:** Comparison of needle sizes 22 and 25 with respect to all variables (7)

Variables	Needle size			*P* value
		22	25	
		N (%)	N (%)	
Adequate		54 (50)	54 (50)	1.000
Inadequate		3 (42.9)	4 (57.1)	
Bloody		8 (61.5)	5 (38.5)	0.359
Non-bloody		49 (48)	53 (52)	
Conclusive		55 (49.1)	57 (50.9)	0.618
Non-conclusive		2 (66.7)	1 (33.3)	
No. of passes	≤3	31 (44.9)	38 (55.1)	0.223
	>3	26 (56.5)	20 (43.5)	

P value <0.05 is statistically significant.

The comparison of needle sizes 22 and 25 in terms of lymph node variables showed that there was no significant difference in adequacy rates, but needle size 22 had a higher incidence of bloody samples, which was statistically significant. There was no significant difference between the two groups in conclusiveness, non-conclusiveness, or the number of passes made during the FNA procedure (*P* > 0.05) (Table 5).

**Table 5:** Comparison of needle sizes 22 and 25 with respect to variables (lymph node)

Variables	Needle size			*P* value
		22	25	
		N (%)	N (%)	
Adequate		20 (54.1)	17 (45.9)	1.000
Inadequate		1 (100)	0 (0)	
Bloody		9 (100)	0 (0)	**0.002**
Non-bloody		12 (41.4)	17 (58.6)	
Conclusive		20 (55.6)	16 (44.4)	1.000
Non-conclusive		1 (50.0)	1 (50.0)	
No. of passes	≤3	18 (60)	12 (40)	0.426
	>3	3 (37.5)	5 (62.5)	

P value <0.05 is statistically significant.

The binary logistic regression analysis was conducted to determine the odds ratios and confidence intervals for the variables predicting adequacy in both thyroid and lymph node FNA. The results show that the number of passes was a significant predictor of adequacy, with an odds ratio of 0.068 (95% confidence interval: 0.008–0.570) and a *P* value of 0.013. This suggests that as the number of passes increases, the odds of obtaining an adequate sample decrease. Neither needle size nor the interaction between needle size and the number of passes was a significant predictor of adequacy (Table 6).

**Table 6:** Binary logistic regression analysis for adequacy (both thyroid and lymph nodes)

Variables	OR	95% CI	*P* value
Needle size × number of passes	0.289	(0.035–2.414)	0.252
Needle size	0.866	(0.198–3.782)	0.848
Number of passes	0.068	(0.008–0.570)	**0.013**

P value <0.05 is statistically significant.

## Discussion

The FNA procedure is commonly used to initially evaluate enlarged thyroid nodules and lymph nodes. It is a quick, cost-effective, minimally invasive, reliable, and practical method. This affordable, straightforward, and painless diagnostic tool plays a crucial role in distinguishing between benign and malignant nodules, providing valuable information for treatment and surgical planning. The primary objective of this study was to compare the rate of cytology specimen adequacy in thyroid nodules and lymph nodes obtained using 22- and 25-gauge needles during FNA. The diagnostic accuracy achieved was 93.6%^[14]^. Several factors can influence the cytological adequacy of ultrasound-guided FNA of thyroid nodules and lymph nodes. These variables include the nodule’s solidity or vascularity, the method of sampling (aspiration or capillary acquisition), the number of samples obtained (single or multiple), the number of needle passes, the use of aspiration devices, and the gauge of the needle^[15–18]^. The selection of the needle is a critical technical aspect of FNA. Both liquid-based cytology and traditional smear cytology are impacted by the gauge of the needle, making it an important consideration. The adequacy of the smear plays a significant role in determining the appropriate needle for the procedure^[19]^.

We examined variables such as sample adequacy, presence of bloody samples, conclusiveness of results, number of passes during the procedure, and the impact of needle size on these factors. It was found that both thyroid and lymph node samples obtained through FNA had high rates of sample adequacy, with 93.9% and 97.4% considered adequate, respectively. This indicates that FNA is generally effective in providing sufficient samples for diagnostic purposes in both types of cases. The incidence of bloody samples was slightly higher in the lymph node group, but overall, the rates were relatively low and not statistically significant. FNA yielded conclusive results in 97.4% of thyroid samples and 94.7% of lymph node samples, demonstrating its reliability in providing definitive diagnoses. The number of passes during the FNA procedure differed significantly between thyroid and lymph node samples, with a higher percentage of lymph node samples requiring more than three passes. This suggests that collecting an adequate sample from lymph nodes may be more challenging due to their anatomical location and size. However, it was previously observed that the thyroid gland is extremely vascular, perhaps resulting in increased hemorrhagic aspirates during FNA. This demands meticulous approach and may take additional passes to acquire sufficient samples. Conversely, lymph nodes have reduced vascularity, which may decrease the probability of blood-contaminated specimens^[8]^. Conversely, the diagnostic efficacy of FNA declines in instances involving tiny lymph nodes, lymphocytic infiltrates or necrosis, and cyst aspirates devoid of epithelial components^[20]^, which may provide low cell counts or inadequate sample representation^[21]^.

As per the 22- and 25-gauge needle comparison, in thyroid samples, there was no significant difference in terms of adequacy, presence of bloody samples, conclusiveness, or the number of passes between the two needle sizes. Similarly, in lymph node samples, there was no significant difference in adequacy, conclusiveness, non-conclusiveness, or the number of passes based on needle size. These results suggest that both needle sizes are comparable in terms of their diagnostic utility.

Furthermore, a binary logistic regression analysis was conducted to determine the predictors of adequacy in both thyroid and lymph node FNA. The number of passes was identified as a significant predictor of adequacy. As the number of passes increased, the odds of obtaining an adequate sample decreased. However, needle size and the interaction between needle size and the number of passes were not significant predictors of adequacy.

In a recent study conducted by Lee *et al*^[22]^, it was found that both 23- and 25-gauge needles are effective when solution-based samples are used in FNA. The study indicated that there were no statistically significant differences in cytological adequacy or complications between the two needle sizes. The research showed that both 23- and 25-gauge needles yielded high rates of cytological adequacy, with percentages of 91.8% and 92%, respectively. They concluded that the cytological adequacy of ultrasound-guided FNA for liquid-based cytology remains unaffected regardless of the needle size used, ranging from 21 to 25 gauge^[22]^. The study conducted by Tangpricha *et al*^[4]^ revealed that while larger needles (21-gauge) may result in higher cellularity during FNA compared to thinner ones (25-gauge), they may not necessarily improve diagnostic accuracy. These results are compatible with our findings.

A separate study discovered that 25-gauge needles exhibited the highest sample quality scores when compared to 22-gauge and 23-gauge needles in thyroid FNA. Based on their findings, the researchers recommended that routine thyroid FNA procedures be conducted using a 25-gauge needle^[19]^. Degirmenci *et al*^[23]^ observed a decrease in the occurrence of inadequate specimens when finer needles (24–25-gauge) were employed. Taking these findings into consideration, there are still no clear recommendations on the needle gauge used for FNA.

The presence of blood contamination in thyroid FNAC can contribute to inadequate cytological results^[24–26]^. A study conducted in Japan revealed that a higher frequency of using 22-gauge needles, as compared to 25-gauge needles was associated with a greater degree of blood contamination^[27]^. As per our results, there was no statistically significant difference between blood contamination in samples obtained using 22-gauge and 25-gauge needles, which yields the fact that the existing evidence is inconsistent.

Limitations of the study include its retrospective design, which relies on data extracted from electronic medical records. This may introduce the possibility of incomplete or inaccurate data as well as the inability to control for certain confounding variables. Additionally, the study was conducted at a single center, which limits the generalizability of the findings to other populations and settings. The sample size was relatively small, which may impact the statistical power and precision of the results. Furthermore, the study focused on comparing two specific needle sizes (22 and 25 gauge) and did not consider other potential factors that could influence sample adequacy and diagnostic accuracy. Therefore, the findings should be interpreted within the context of these limitations, and further research is needed to validate the results in larger and more diverse populations.

## Conclusion

The study highlights the importance of the number of passes during the procedure, with a higher number of passes associated with decreased odds of obtaining an adequate sample. The needle size does not significantly impact the various variables assessed. Further large-scale studies are needed to establish clear recommendations on the usage of 22-gauge and 25-gauge needles for thyroid FNA.

## Data Availability

The datasets supporting the conclusions of this article are included within the article.

## Research registration unique identifying number (UIN)

UIN: researchregistry10797 (https://www.researchregistry.com/browse-theregistry#home/registrationdetails/671e37931ecab802fc1389dc).

## References

[1] Hegedüs L. Clinical practice. The thyroid nodule. N Engl J Med 2004;351:1764–71. 15496625 10.1056/NEJMcp031436

[2] Alexander EK Doherty GM Barletta JA. Management of thyroid nodules. Lancet Diabetes Endocrinol 2022;10:540–48. 35752201 10.1016/S2213-8587(22)00139-5

[3] Borson-Chazot F Buffet C Decaussin-Petrucci M. SFE-AFCE-SFMN 2022 consensus on the management of thyroid nodules: synthesis and algorithms. Ann Endocrinol (Paris) 2022;83:440–53. 36336101 10.1016/j.ando.2022.11.001

[4] Tangpricha V Chen BJ Swan NC. Twenty-one-gauge needles provide more cellular samples than twenty-five-gauge needles in fine-needle aspiration biopsy of the thyroid but may not provide increased diagnostic accuracy. Thyroid 2001;11:973–76. 11716046 10.1089/105072501753211055

[5] Halenka M Fryšák Z. Atlas of thyroid ultrasonography. Halenka M Fryšák Z, eds. Ultrasound-Guided Fine-Needle Aspiration Biopsy (US-FNAB). Springer International Publishing; 2017:383–92.

[6] Can AS Peker K. Comparison of palpation-versus ultrasound-guided fine-needle aspiration biopsies in the evaluation of thyroid nodules. BMC Res Notes May 15 2008;1:12. 18710537 10.1186/1756-0500-1-12 PMC2518275

[7] Kim DW. Ultrasound-guided fine-needle aspiration for retrojugular lymph nodes in the neck. World J Surg Oncol May 30 2013;11:121. 23721570 10.1186/1477-7819-11-121 PMC3671968

[8] Xia S Chen Y Zhan W. Ultrasound-guided fine-needle aspiration versus fine-needle capillary sampling in evaluation of lymph node metastasis of thyroid cancer. Front Oncol 2021;11:642142. 33937044 10.3389/fonc.2021.642142 PMC8079778

[9] Cooper DS Doherty GM Haugen BR. Revised American Thyroid Association management guidelines for patients with thyroid nodules and differentiated thyroid cancer. Thyroid 2009;19:1167–214. 19860577 10.1089/thy.2009.0110

[10] Cao C Jasim S Cherian A. Patient discomfort in relation to thyroid nodule fine-needle aspiration (FNA) performed with or without parenteral and/or topical anesthetic. Endocr Pract 2020;26:1497–504. 33471742 10.4158/EP-2020-0403

[11] Grani G Calvanese A Carbotta G. Intrinsic factors affecting adequacy of thyroid nodule fine-needle aspiration cytology. Clin Endocrinol (Oxf) 2013;78:141–44. 22812685 10.1111/j.1365-2265.2012.04507.x

[12] Shah KS Ethunandan M. Tumour seeding after fine-needle aspiration and core biopsy of the head and neck–a systematic review. Br J Oral Maxillofac Surg 2016;54:260–65. 26837638 10.1016/j.bjoms.2016.01.004

[13] Mathew G Agha R Albrecht J. STROCSS 2021: strengthening the reporting of cohort, cross-sectional and case-control studies in surgery. Int J Surg 2021;96:106165. 34774726 10.1016/j.ijsu.2021.106165

[14] Sharma C. Diagnostic accuracy of fine needle aspiration cytology of thyroid and evaluation of discordant cases. J Egypt Natl Canc Inst 2015;27:147–53. 26185872 10.1016/j.jnci.2015.06.001

[15] Fadda G Rossi ED. Liquid-based cytology in fine-needle aspiration biopsies of the thyroid gland. Acta Cytol 2011;55:389–400. 21986164 10.1159/000329029

[16] Nagarajan N Schneider EB Ali SZ. How do liquid-based preparations of thyroid fine-needle aspiration compare with conventional smears? An analysis of 5475 specimens. Thyroid 2015;25:308–13. 25420135 10.1089/thy.2014.0394

[17] Abraham TM de Las Morenas A Lee SL. In thyroid fine-needle aspiration, use of bedside-prepared slides significantly increased diagnostic adequacy and specimen cellularity relative to solution-based samples. Thyroid 2011;21:237–42. 21323589 10.1089/thy.2010.0211

[18] Jung SJ Kim DW Baek HJ. Comparison study of the adequacy and pain scale of ultrasound-guided fine-needle aspiration of solid thyroid nodules with a 21- or 23-gauge needle for liquid-based cytology: a single-center study. Endocr Pathol 2018;29:30–34. 29275502 10.1007/s12022-017-9508-1

[19] Dong Y Gao L Sui Y. Comparison of ultrasound-guided fine-needle cytology quality in thyroid nodules with 22-, 23-, and 25-gauge needles. Anal Cell Pathol (Amst) 2021;2021:5544921. 34211823 10.1155/2021/5544921 PMC8205598

[20] Jiang HJ Hsiao PJ. Clinical application of the ultrasound-guided fine needle aspiration for thyroglobulin measurement to diagnose lymph node metastasis from differentiated thyroid carcinoma-literature review. Kaohsiung J Med Sci 2020;36:236–43. 31909556 10.1002/kjm2.12173 PMC11896569

[21] Orija IB Hamrahian AH Reddy SS. Management of nondiagnostic thyroid fine-needle aspiration biopsy: survey of endocrinologists. Endocr Pract 2004;10:317–23. 15760774 10.4158/EP.10.4.317

[22] Lee YJ Kim DW Shin GW. Comparison of cytological adequacy and pain scale score in ultrasound-guided fine-needle aspiration of solid thyroid nodules for liquid-based cytology with with 23- and 25-gauge needles: a single-center prospective study. Sci Rep 2019;9:7027. 31065031 10.1038/s41598-019-43615-7 PMC6504848

[23] Degirmenci B Haktanir A Albayrak R. Sonographically guided fine-needle biopsy of thyroid nodules: the effects of nodule characteristics, sampling technique, and needle size on the adequacy of cytological material. Clin Radiol 2007;62:798–803. 17604771 10.1016/j.crad.2007.01.024

[24] Polyzos SA Anastasilakis AD. Clinical complications following thyroid fine-needle biopsy: a systematic review. Clin Endocrinol (Oxf) 2009;71:157–65. 19170717 10.1111/j.1365-2265.2009.03522.x

[25] Redman R Zalaznick H Mazzaferri EL. The impact of assessing specimen adequacy and number of needle passes for fine-needle aspiration biopsy of thyroid nodules. Thyroid 2006;16:55–60. 16487014 10.1089/thy.2006.16.55

[26] Cappelli C Pirola I Castellano M. Fine needle cytology of complex thyroid nodules. Eur J Endocrinol 2007;157:529–32. 17893269 10.1530/EJE-07-0172

[27] Tanaka A Hirokawa M Higuchi M. Optimal needle size for thyroid fine needle aspiration cytology. Endocr J Feb 28 2019;66:143–47. 30464152 10.1507/endocrj.EJ18-0422

